# A Study of Al_2_O_3_/MgO Composite Films Deposited by FCVA for Thin-Film Encapsulation

**DOI:** 10.3390/ma16051955

**Published:** 2023-02-27

**Authors:** Heng Yuan, Yifan Zhang, Qian Li, Weiqing Yan, Xu Zhang, Xiao Ouyang, Xiaoping Ouyang, Lin Chen, Bin Liao

**Affiliations:** 1 College of Nuclear Science and Technology, Beijing Normal University, Beijing 100875, China; 2 Advanced Institute of Natural Sciences, Beijing Normal University at Zhuhai, Zhuhai 519087, China

**Keywords:** FCVA, Al_2_O_3_, MgO, thin-film encapsulation

## Abstract

Al_2_O_3_ and MgO composite (Al_2_O_3_/MgO) films were rapidly deposited at low temperatures using filtered cathode vacuum arc (FCVA) technology, aiming to achieve good barrier properties for flexible organic light emitting diodes (OLED) thin-film encapsulation (TFE). As the thickness of the MgO layer decreases, the degree of crystallinity decreases gradually. The 3:2 Al_2_O_3_:MgO layer alternation type has the best water vapor shielding performance, and the water vapor transmittance (WVTR) is 3.26 × 10^−4^ g·m^−2^·day^−1^ at 85 °C and 85% R.H, which is about 1/3 of that of a single layer of Al_2_O_3_ film. Under the action of ion deposition, too many layers will cause internal defects in the film, resulting in decreased shielding ability. The surface roughness of the composite film is very low, which is about 0.3–0.5 nm depending on its structure. In addition, the visible light transmittance of the composite film is lower than that of a single film and increases with the increase in the number of layers.

## 1. Introduction

Organic light emitting diodes (OLED) display technology is being widely studied and used due to a series of advantages such as low energy consumption, light and thin body, bendable, self-illumination, fast response time, and large viewing angle [1]. However, as OLED is vulnerable to water vapor and oxygen erosion, flexible OLED encapsulation has become a big problem to be solved [2]. At present, thin-film encapsulation (TFE) technology is an effective means to extend the service life of flexible OLED [3,4].

At present, most studies on encapsulated film deposition methods focus on ALD technology, which can uniformly form films [3,4]. However, due to its low deposition energy, it is difficult to ensure the high density of deposited films. The deposition process requires repeated gas purging, which leads to slow deposition speed. At the same time, high deposition temperature and residual hydrogen will have a negative impact on OLED and thin-film transistor (TFT) [5,6,7,8]. Filtered cathode vacuum arc (FCVA) technology has the advantages of fast deposition, low deposition temperature, high film density, and no hydrogen in the deposition process, so it has potential in the field of TFE [9,10]. In our previous work, we have demonstrated that single-layer 100 nm Al_2_O_3_ films prepared using FCVA technology can effectively extend the service life of OLED devices, and the advantages of low internal stress and high adhesion make the films suitable for flexible encapsulation [11,12]. Some reports indicate that the failure of Al_2_O_3_ films will be accelerated by hydrolysis in the face of high temperature and high humidity [13]. The defects of inorganic films exist inevitably in the deposition process, including displacement damage, nano-scale pores, grain boundaries, and local defects caused by impurities [14,15]. The defects in the film tend to grow continuously, and water molecules can easily penetrate along the defects to reach the interface between the film and the substrate. This will not only corrode the substrate but also reduce the adhesion between the film and the substrate and accelerate the failure of the film. Although films of different thicknesses can prolong the permeation path of water vapor, once water molecules break through the permeation channel, the whole film still faces the risk of failure. Some studies also show that the barrier capacity of inorganic films does not linearly increase with their thickness [16,17].

The design of a multilayer inorganic composite film stack can prevent the formation of pores but also help to prevent the continuous growth of defects in the vertical direction of the film [18]. In addition, if the material with strong water absorption ability is inside the film, it can effectively delay the further erosion of water vapor [19]. Many researchers have found that the barrier performance of inorganic layers is improved when inorganic thin films are alternately stacked for TFE [20,21,22,23].

With a wide surface band gap width (E_g_^S^ = 6.3 eV) and bulk band gap width (E_g_^B^ = 7.8 eV) and outstanding chemical stability, magnesium oxide (MgO) has potential applications in TFE [24,25,26]. In this paper, FCVA technology was used to explore the MgO film, and a variety of structures of composite Al_2_O_3_/MgO film was designed. The main purpose of this is to use MgO intercalation to avoid the continuous growth of internal defects in Al_2_O_3_ so as to improve the penetration path of water vapor. In addition, taking advantage of the moisture absorption characteristics of MgO, it is used as an internal absorption layer to assist Al_2_O_3_ film in blocking water vapor. The structure and properties of the composite film are analyzed in this paper.

## 2. Materials and Methods

**Figure 1:**
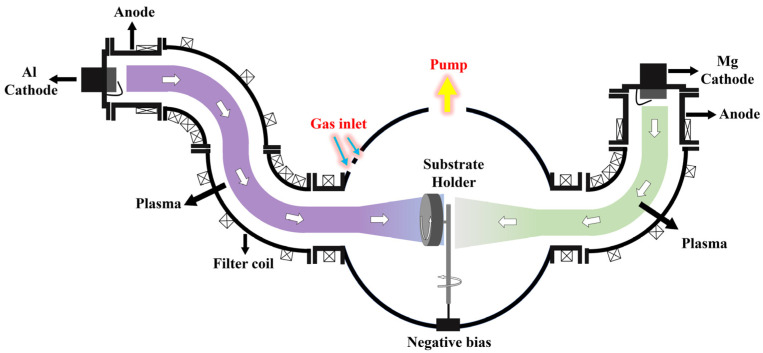
Schematic diagram of FCVA equipment used for deposition.

Polyethylene naphthalate (PEN) (50 μm, Teijin Limited, Tokyo, Japan) and silicon (100) substrates were ultrasonically cleaned for 15 min with acetone, ethanol, and deionized water, respectively, prior to initiation of deposition. After ultrasonic cleaning, the substrate was dried with nitrogen and then deposited in a vacuum chamber. The Mg target was used as a cathode source to deposit MgO, and neutral particles and metal droplets were filtered by a 90° magnetic filter. Al_2_O_3_ was deposited using an Al target as a cathode source, and neutral particles and metal droplets were filtered using a 180° magnetic filter. A schematic diagram of the depositional equipment is shown in Figure 1. The vacuum chamber was pumped as low as 4.0 × 10^−3^ pa, and the cathode was excited by an arc current of 60 A. During deposition, 8 sccm argon and 4 sccm oxygen were introduced into the vacuum chamber. Negative bias was introduced on the substrate at a frequency of 50 Hz and a pulse width of 4 μs. The deposition rates of Al_2_O_3_ and MgO are about 15 nm/min and 37 nm/min, respectively. The thickness of all films is about 100 nm.

A surface profilometer (Talysurf 5P-120, Taylor Hobson, Leicester, UK) was used to measure the thickness of the thin-film samples. The surface and cross-section morphology of the samples were observed by cold field emission scanning electron microscopy (SEM, S-4800, Hitachi, Tokyo, Japan). Atomic force microscopy (AFM, Tosca™400, Anton Pal, Austria) is used to characterize surface roughness. The Infrared transmission spectrum (FT-IR, iS20, Thermo Scientific Nicolet, Graz, Waltham, MA, USA) test was conducted in ATR (attenuated total reflectance mode), and the test range was 500–4000 cm^−1^. The chemical composition of the sample was characterized using X-ray photoelectron spectroscopy (XPS, ESCALAB 250Xi, Thermo Fisher Scientific, Waltham, MA, USA). Before the XPS test, Ar^+^ sputtering etched the sample, and the binding energy was calibrated by c1s peak (284.4 eV). Transmission electron microscopy (TEM, g20, FEI, Hillsboro, USA) was used to analyze the microstructure of the films. TEM samples of the test film were prepared on the NaCl substrate, and then the NaCl substrate was dissolved with distilled water, and the suspension was collected using a microgrid with a carbon support film for TEM testing. X-ray Diffraction (XRD, D8 Advance, Bruker, Karlsruhe, Germany) was employed to analyze the microstructure of the films. The transmittance of the film in the visible range was measured by an ultraviolet-visible spectrophotometer (SPECORD200, Carl Zeiss AG, Oberkochen, Germany). The corrosion test of MgO film was carried out using a programmable constant temperature and humidity test chamber (QL-HWHS-80L, QunLong, Guangzhou, China). The test environment was 85 °C and 85% relative humidity (R.H). The water vapor transmittance (WVTR) of the samples was determined by the Mg method [11,12]. The devices used in the Mg test method were prepared by the following methods: First, the Al film with a thickness of 400 nm was deposited on both sides of the glass substrate using FCVA technology as the electrode, and then the Mg film with a thickness of 270 nm and an area of 4 × 4 cm^2^ was deposited between the electrodes. After that, the sample to be tested was sealed on the Mg film with UV-cured resin (K2018, Ksimi, Shenzhen, China). The device was placed in the programmable constant temperature and humidity test chamber (QL-HWHS-80L, QunLong, Guangzhou, China) to control the environment at 85 °C and 85% R.H. The real-time change of Mg film resistance was monitored with a four-probe resistance tester (FT-430, Rooko, Ningbo, China). The reaction of Mg with high-temperature water vapor will produce insulator magnesium hydroxide (Mg(OH)_2_). Therefore, the WVTR of the film at high temperatures can be calculated by monitoring the conductivity of the Mg film.

## 3. Results

**Figure 2:**
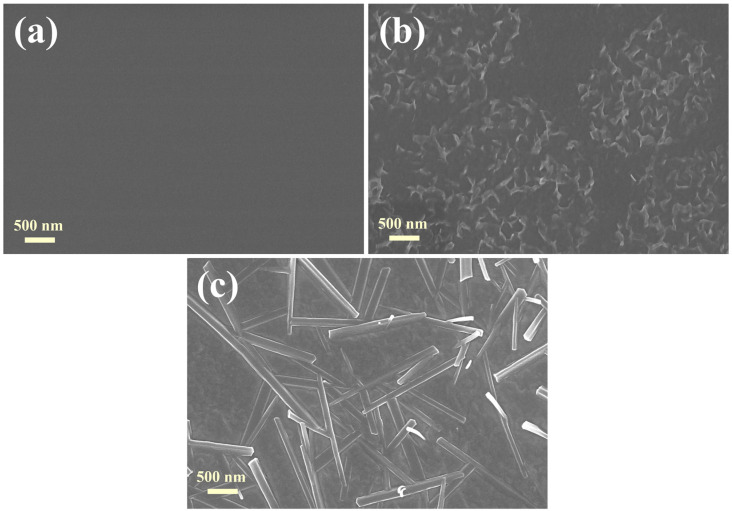
SEM surface morphologies of MgO films placed at 85 °C, 85% R.H for different times: (**a**) 0 h, (**b**) 6 h, and (**c**) 12 h.

MgO films deposited by physical vapor deposition (PVD) usually have large internal stress, which will lead to poor film quality and even cannot grow into films [27]. Our experiments have proved that the MgO film deposited without negative bias is in the form of powder. The internal stress of the film can be effectively reduced by introducing high-power negative pulsed bias [11]. After the negative bias voltage of 7 kV was applied, the MgO film was successfully grown. In order to explore the reaction of MgO thin film with water vapor in a high temperature and high humidity environment, a corrosion test was carried out, the thickness of the thin film was 100 nm, and the deposition substrate was Si. The change results of its surface morphology are shown in Figure 2. As can be seen from the figure, the MgO film before the corrosion test shows a smooth and flat surface topography, which indicates that the deposition of large particles can be effectively avoided by filtering through the 90° magnetic filter. After 6 h of the experiment, typical petal-like hydrolytic morphology can be seen on the surface of the MgO film, as shown in Figure 2b. After the corrosion experiment for 12 h, a large number of fibrous crystals appeared on the surface of the film, the length of which was about 1–2 μm, which is Mg(OH)_2_ generated by the reaction of MgO with water vapor. The reaction equation of MgO with water is shown as follows [28]:

(1) $$\begin{array}{c} MgO+H_{2}O=Mg({OH)}_{2} \end{array}$$

**Figure 3:**
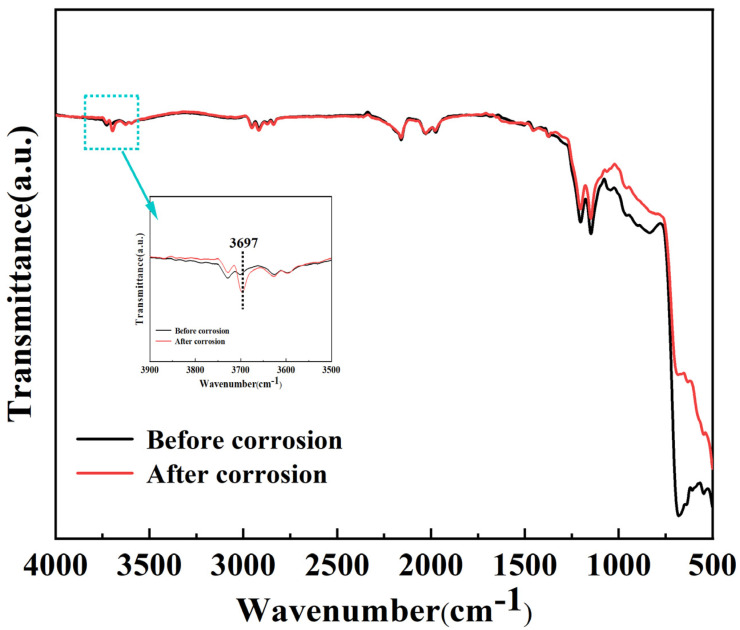
FT-IR spectra of MgO film.

To explore the structural changes of MgO films before and after corrosion, FT-IR was employed to test the samples. The FT-IR spectra of MgO film before and after corrosion are shown in Figure 3. It can be seen from the figure that the corroded MgO film has an absorption peak at 3697 cm^−1^, which is due to the OH group in Mg(OH)_2_ [29]. The bands in the region below 1000 cm^−1^ are derived from an overtone of a fundamental lattice vibration of Mg-O stretching. The absorption intensity of the corroded MgO film in this region is significantly reduced, which proves that MgO is consumed by the corrosion reaction [30,31]. There is no obvious absorption peak at 1640 cm^−1^, which corresponds to the bending vibration of the adsorbed water, and no wide lines corresponding to the O-H bonds of adsorbed water are observed in the range of 3000–4000 cm^−1^, indicating that the sample is fully dried [32,33]. The test results of FT-IR further confirmed that MgO would generate Mg(OH)_2_ after water vapor corrosion.

**Figure 4:**
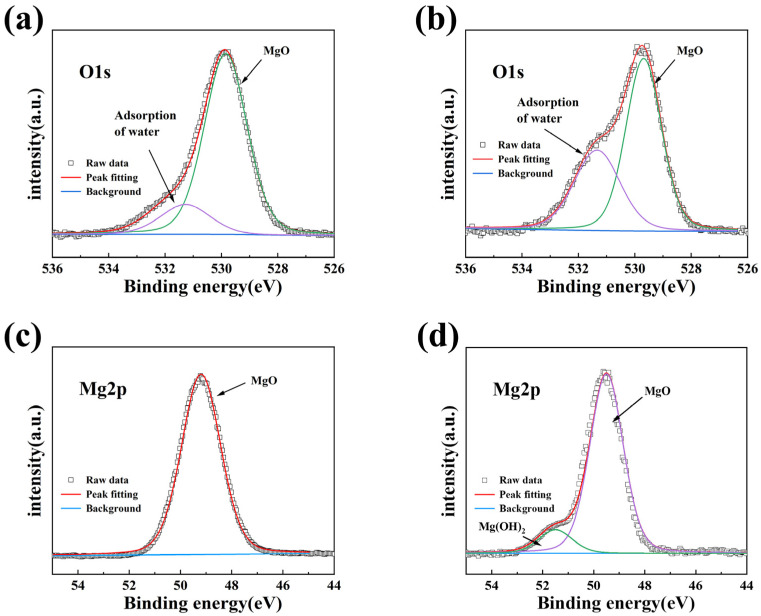
O 1s XPS spectra of MgO film: (**a**) before corrosion, (**b**) after corrosion; Mg 2p XPS spectra of MgO film: (**c**) before corrosion and (**d**) after corrosion.

To further explore the corrosion performance of MgO films in high temperature and high humidity environment, XPS analysis was conducted on MgO films before and after corrosion. O 1s and Mg 2p XPS spectra of MgO films are shown in Figure 4. The O 1s high-resolution spectrum of the deposited MgO film showed two peaks, located at 529.8 eV and 531.4 eV, respectively, representing MgO and the adsorption of water, indicating that MgO has a strong adsorption capacity for water molecules in the air [28]. After 6 h of corrosion, the proportion of the peak area of water adsorption in O1s increased significantly, indicating that the adsorption capacity of MgO on water molecules in high temperature and high humidity environment was further strengthened. The Mg 2p XPS spectra show that the MgO film only has a single peak at 49.7 eV before corrosion, which represents the Mg-O bond in MgO [19]. After corrosion, a new peak appeared at 50.8 eV, representing Mg ions in the hydroxide matrix, indicating that MgO degraded and began to react with water vapor to form Mg(OH)_2_ [19]. Based on the above analysis, it can be seen that MgO is not suitable for the most single-layer water vapor shield film, mainly because of its lack of stability in the face of water vapor and easy hydrolysis reaction. However, the moisture absorption ability of MgO film makes it possible to act as an internal moisture absorption layer.

**Figure 5:**
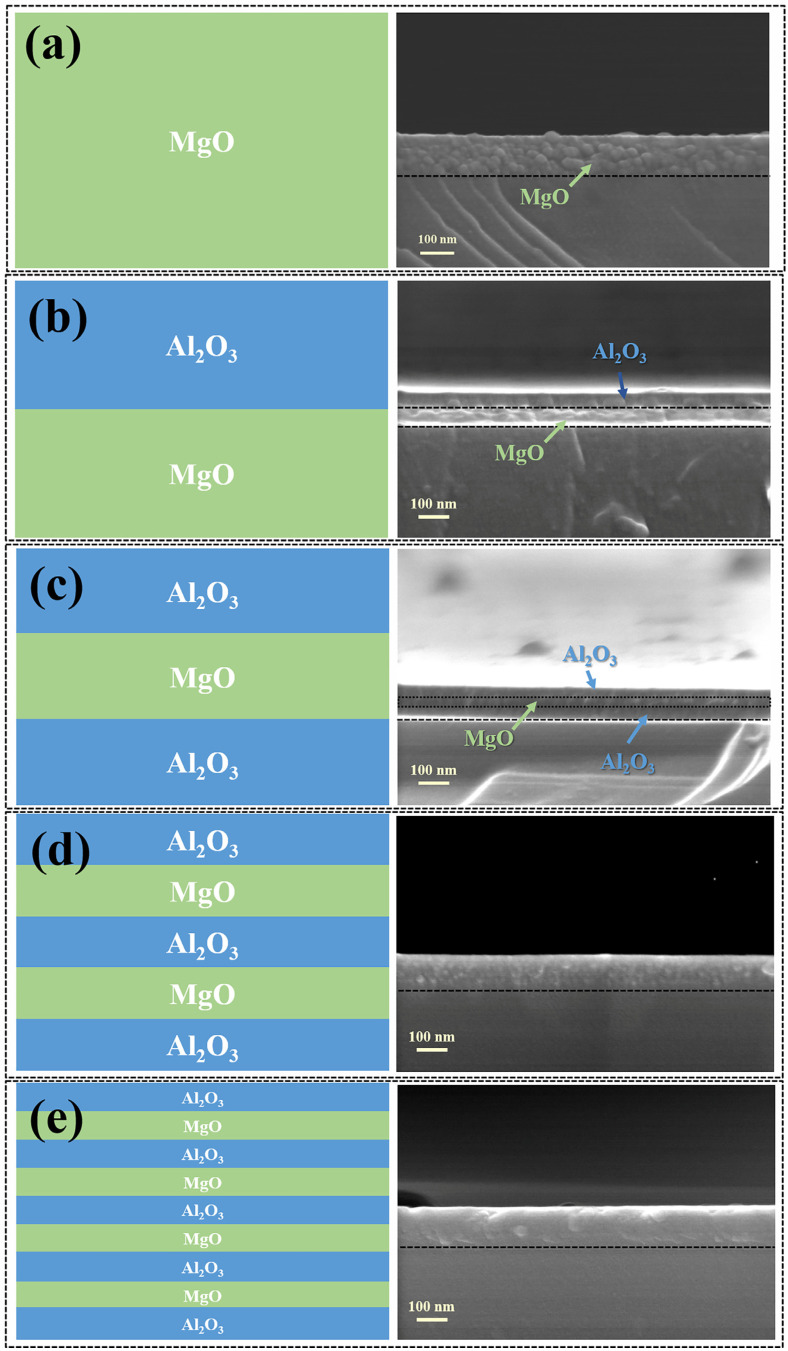
Structure diagram and cross-section SEM morphology of 100 nm thin film: (**a**) MgO, (**b**) 1:1 Al_2_O_3_:MgO layer alternation type, (**c**) 2:1 Al_2_O_3_:MgO layer alternation type, (**d**) 3:2 Al_2_O_3_:MgO layer alternation type, and (**e**) 5:4 Al_2_O_3_:MgO layer alternation type.

We further designed and prepared a variety of Al_2_O_3_/MgO composite films. SEM was used to characterize the cross-section morphology of MgO thin films and a variety of Al_2_O_3_/MgO composite thin films. The results are shown in Figure 5. It can be seen from Figure 5a that the cross-section of MgO film presents a large number of continuous tightly packed spherical clusters. The cluster particles can weaken the internal compression of the film caused by the surface tension of the substrate, which is conducive to the release of the internal stress of the film [34]. As can be seen from Figure 5b, the MgO part of the 1:1 Al_2_O_3_:MgO layer alternation type is clearly distinguished from the Al_2_O_3_ part, and MgO presents an obvious equiaaxial crystal structure, while Al_2_O_3_ has no obvious morphological characteristics. It can be seen from Figure 5c that MgO crystalline particles can still be observed in the middle section of the 2:1 Al_2_O_3_:MgO layer alternation type. As can be seen from Figure 5d, due to the further thinning of MgO in the 3:2 Al_2_O_3_:MgO layer alternation type, a large number of fine cluster particles appear inside the film, which may be due to the alternating Al_2_O_3_ interrupting the growth of MgO grains. In Figure 5e, the cross-section of the 5:4 Al_2_O_3_:MgO layer alternation type presents a uniform and dense structure, and there is no obvious MgO spherical grain.

**Figure 6:**
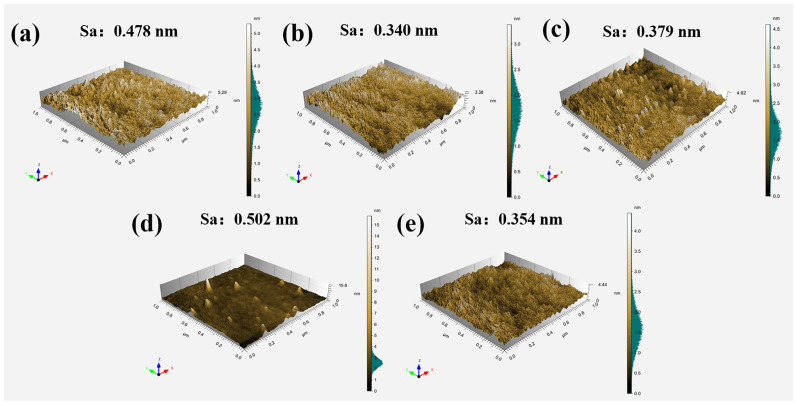
AFM surface morphology of 100 nm thin film: (**a**) MgO, (**b**) 1:1 Al_2_O_3_:MgO layer alternation type, (**c**) 2:1 Al_2_O_3_:MgO layer alternation type, (**d**) 3:2 Al_2_O_3_:MgO layer alternation type, and (**e**) 5:4 Al_2_O_3_:MgO layer alternation type.

AFM was used to characterize the surface morphology of MgO films and various Al_2_O_3_/MgO composite films, and the results are shown in Figure 6. As can be seen from Figure 6a, the surface roughness of MgO film is 0.478 nm. In Figure 6b, the surface roughness of 1:1 Al_2_O_3_:MgO layer alternation type is 0.34 nm, which is lower than that of MgO thin film because the roughness of thin film is mainly dominated by Al_2_O_3_ with a surface thickness of 50 nm. Figure 6c shows that the surface roughness of the 2:1 Al_2_O_3_:MgO layer alternation type rises to 0.379 nm. This is because the thickness of Al_2_O_3_ on the surface decreases, and the surface roughness begins to be affected by internal MgO crystallization. In Figure 6d, the AFM result of the 3:2 Al_2_O_3_:MgO layer alternation type shows that its roughness is 0.502 nm, and there are columnar particles on the film surface, which indicates that the surface roughness of the film is affected by the internal MgO grains with the further reduction of the surface Al_2_O_3_ thickness. However, with the further increase in the number of composite film layers, it can be seen from Figure 6e that the surface roughness of the 5:4 Al_2_O_3_:MgO layer alternation type decreased to 0.354 nm.

**Figure 7:**
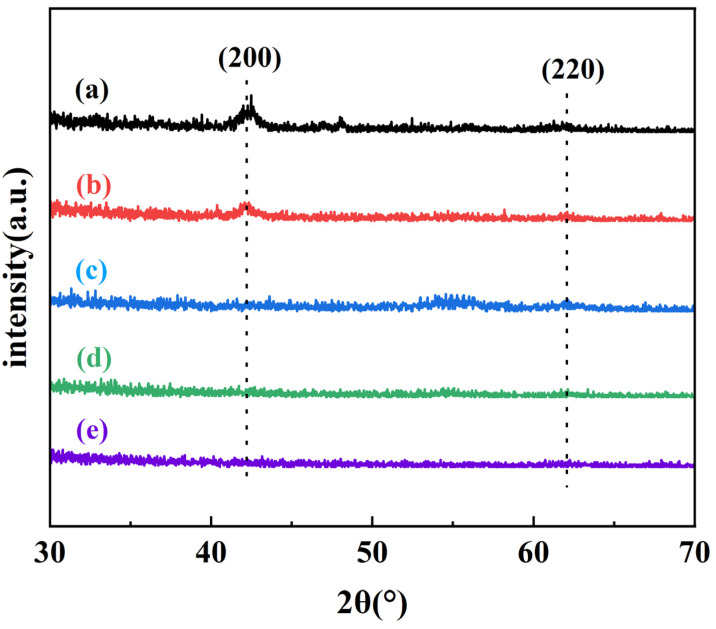
XRD patterns of 100 nm thin film: (**a**) MgO, (**b**) 1:1 Al_2_O_3_:MgO layer alternation type, (**c**) 2:1 Al_2_O_3_:MgO layer alternation type, (**d**) 3:2 Al_2_O_3_:MgO layer alternation type, and (**e**) 5:4 Al_2_O_3_:MgO layer alternation type.

To further explore the structure of Al_2_O_3_/MgO composite films with different structures, the samples were tested by XRD, and the results are shown in Figure 7. It can be seen that MgO has diffraction peaks at 42.8 ° and 62.2 °, representing MgO (200) and MgO (220) planes, respectively, which is consistent with cubic MgO. The experimental data are consistent with the International Center for Diffraction Data (Newtown Square, PA). The diffraction peak of MgO (200) can be found in the XRD results of the 1:1 Al_2_O_3_:MgO layer alternation type, but the intensity has been significantly weakened. With the further refinement of the composite film structure, no obvious diffraction peaks were observed in the 2:1 Al_2_O_3_:MgO, 3:2 Al_2_O_3_:MgO, and 5:4 Al_2_O_3_:MgO layer alternation type, indicating that the degree of the crystallinity became lower, which was consistent with the observation of the SEM test results above. In addition, Al_2_O_3_ does not exhibit any diffraction peak, indicating that Al_2_O_3_ is amorphous, which is consistent with our previous research [11].

**Figure 8:**
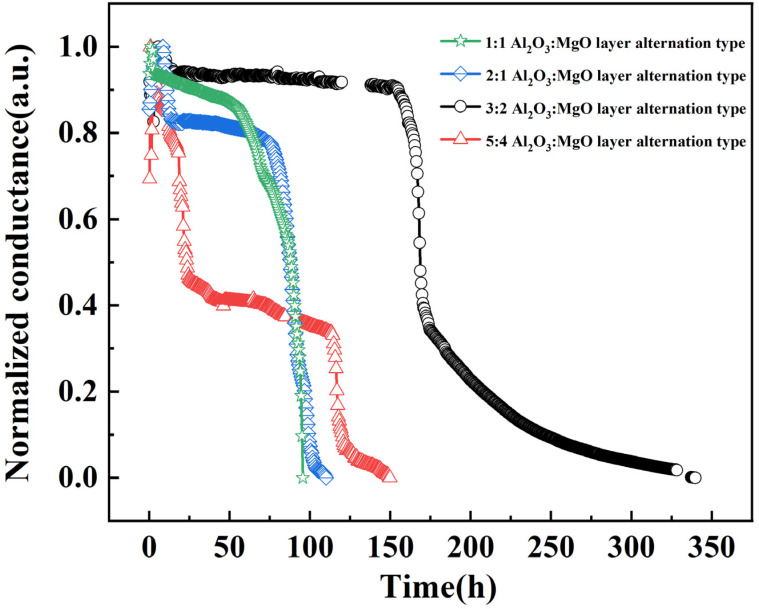
Mg test results of films with different composite structures.

To obtain the water vapor barrier performance of composite films, the WVTR of composite films with several structures was tested. The conductivity decline curve of Mg film under thin-film encapsulation is shown in Figure 8. The Mg film reacts with high-temperature water vapor to produce non-conductive Mg(OH)_2_, so its conductivity gradually declines with the test. At the beginning of the experiment, the conductivity of Mg increased by a short period. We think this is the fluctuation caused by the poor contact between the device and the four-probe resistance tester at the beginning of the test. After that, the conductivity of the Mg film decreased rapidly, which we believe is due to the reaction between the adsorbed water in the device and the Mg film. Then, the conductivity of Mg film enters a stable stage, which is called Lag time [17]. During this period, water molecules penetrated into the test film but did not reach the Mg film. After this stage, the water molecules pass through the test film and react with Mg, so the conductivity of Mg rapidly declines to 0. It is worth noting that the slope of Mg conductivity attenuation in the final stage is different for different samples. This is because the corrosion of Mg film is not completely uniform during the actual experiment. The linear change part of the curve was linearly fitted, and the WVTR of the film was calculated using the following formula, which is similar to the Ca test method [35,36]:

(2) $$\begin{array}{c} WVTR=-n\frac{M\left(H_{2}O\right)}{M\left(\mathrm{Mg}\right)}\delta \rho \frac{L}{W}\frac{d1/R}{dt} \end{array}$$

where *δ* is the resistivity of Mg (4.45 × 10^−8^ Ω m), *L* is the length of Mg film, *W* is the width of Mg film, and *R* is the resistance of Mg film. The *n* is the molar equivalent of water vapor participating in the reaction, and its value is 2. In this equation, *M* (H_2_O) is the molar mass of water, *M* (Mg) is the molar mass of Mg, ρ is the density of Mg (1.74 g/cm^3^), and *t* stands for time.

According to the calculation, the WVTR of 1:1 Al_2_O_3_:MgO, 2:1 Al_2_O_3_:MgO, 3:2 Al_2_O_3_:MgO and 5:4 Al_2_O_3_:MgO layer alternation type at 85 °C and 85% R.H is 3.57 × 10^−3^ g·m^−2^·day^−1^, 7.04 × 10^−4^ g·m^−2^·day^−1^, 3.26 × 10^−4^ g·m^−2^·day^−1^ and 8.921 × 10^−4^ g·m^−2^·day^−1^, respectively. According to our previous work, a single 100 nm Al_2_O_3_ film prepared by FCVA has a WVTR of 9.897 × 10^−4^ g·m^−2^·day^−1^ at 85 °C and 85% R.H. [12]. It can be seen from the results that the WVTR of the 1:1 Al_2_O_3_:MgO layer alternation type is higher than that of the single Al_2_O_3_ film, which may be because the water vapor reacts with the bottom MgO after passing through the top Al_2_O_3_ film, resulting in spalling damage of the entire membrane. With the further increase in the number of layers, the WVTR of the film decreases first and then increases. When the composite structure is 3:2 Al_2_O_3_:MgO layer alternation type, the WVTR of the film reaches the lowest value, and the Lag time of the conductivity is significantly increased, which indicates that the lamination structure effectively prolongs the water vapor permeation path [17]. The increase in the WVTR of the 5:4 Al_2_O_3_:MgO layer alternation type film may be due to the low thickness of Al_2_O_3_ and MgO in the structure and subsequently deposited particles are easy to be injected into the previously deposited film and cause damage defects. The WVTR experiment shows that the water vapor barrier ability of Al_2_O_3_ can be effectively improved by adding a MgO layer inside. The film with excellent water vapor barrier ability can effectively prevent the internal metal cathode corrosion or organic layer hydrolysis of OLED and has the possibility of being used for OLED thin-film encapsulation.

**Figure 9:**
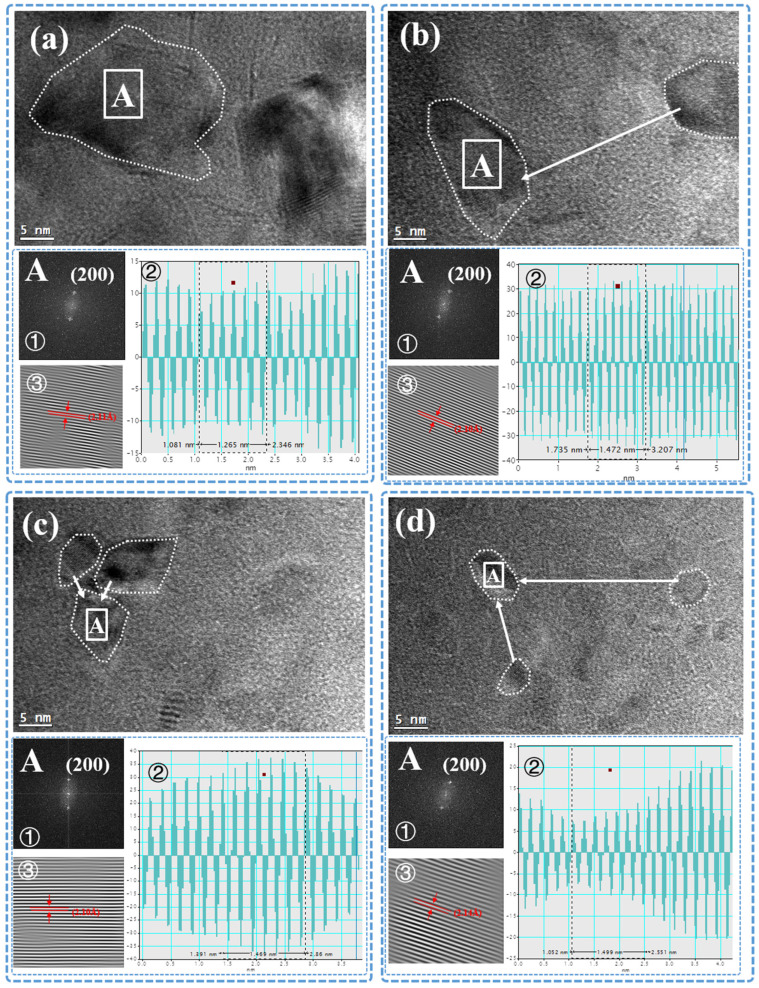
TEM high-resolution (HRTEM) images of different composite films: (**a**) 1:1 Al_2_O_3_:MgO layer alternation type, (**b**) 2:1 Al_2_O_3_:MgO layer alternation type, (**c**) 3:2 Al_2_O_3_:MgO layer alternation type, and (**d**) 5:4 Al_2_O_3_:MgO layer alternation type.

Figure 9 shows TEM high-resolution images of composite films with different structures. The main images are HRTEM images, in which the area indicated by the white arrow is the same crystal face. The area A in the main image is analyzed by using the digital micrograph software package (GMS 3, Gatan Microscopy, Pleasanton, CA, USA), and the analysis results are shown in the sub-image A. In the sub-image, ① is the Fast Fourier Transformations (FFT), ② is profile of Inverse Fast Fourier Transform (IFFT) and ③ is IFFT images. According to the measurement, the interplanar spacing of the MgO crystal (200) plane in 1:1 Al_2_O_3_:MgO, 2:1 Al_2_O_3_:MgO, 3:2 Al_2_O_3_:MgO, and 5:4 Al_2_O_3_:MgO layer alternation type is 2.11Å, 2.10 Å, 2.10 Å, and 2.14 Å, respectively. Compared with the interplanar spacing of the MgO standard lattice (200) at 2.106 Å (according to ICSD card number 98-015-9376, d_200_ = 2.106), the crystal interplanar spacing in the 5:4 Al_2_O_3_:MgO layer alternation type is significantly increased, which further indicates that the MgO lattice in the 5:4 Al_2_O_3_:MgO layer alternation type is damaged by the subsequent deposition of Al and O ions. The growth mode of the ion-deposited film is the subplantation model, so under the influence of negative bias, the subsequently deposited ions will be further injected into the previously deposited film, causing damage defects in its interior [37]. The thin film may form the defect of penetrating up and down, accelerating the infiltration of water vapor. This explains why the ability of the 5:4 Al_2_O_3_:MgO layer alternation type to shield water vapor is reduced.

**Figure 10:**
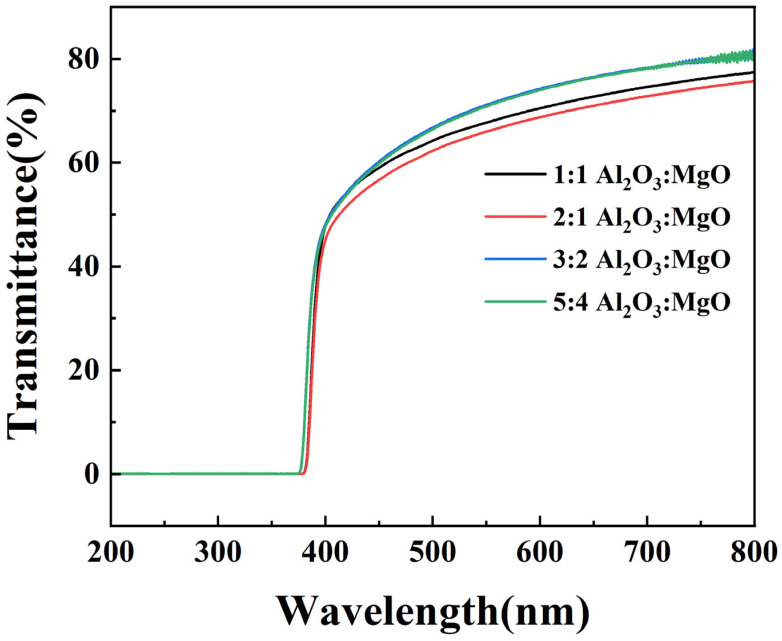
Visible light transmittance of Al_2_O_3_/MgO composite films with different layer alternation types and total thickness of 100 nm on PEN substrate.

In order to explore the transmittance of composite films with different layer alternation types in the visible range, the samples were tested with an ultraviolet-visible spectrophotometer, and the results are shown in Figure 10. In the visible range (400–800 nm), compared with the simple Al_2_O_3_ film (the transmittance is >85%), the transmittance of several composite films is decreased, mainly because the interface layer between Al_2_O_3_ and MgO may produce scattering problems, so it is difficult to obtain the transmittance value of a single film [12]. However, the transmittance of the composite film remains relatively high, considering that the PEN substrate has a transmittance of approximately 82%. Compared with 1:1 Al_2_O_3_:MgO and 2:1 Al_2_O_3_:MgO layer alternation type, the transmittance of 5:4 Al_2_O_3_:MgO and 3:2 Al_2_O_3_:MgO layer alternation type composite films is greatly improved, which is mainly due to the decrease in MgO crystallinity inside the films. The crystalline structure can lead to light scattering, so the light transmittance of a film with low crystallinity is higher than that of a film with high crystallinity [22]. In addition, the internal low crystallinity MgO will reduce the roughness at the interface, which will lead to a decrease in optical scattering and also increase the transmittance [21,38]. In the region of 200–380 nm, the optical transmittance of several composite films is extremely low, which may be due to the point structure defects (F-centers or oxygen vacancies with trapped electrons) of Al_2_O_3_ and MgO having absorption bands in this region [15,39].

## 4. Conclusions

In this paper, a variety of Al_2_O_3_/MgO composite films are prepared using FCVA technology, which can be used for OLED film packaging. MgO film has excellent hygroscopic properties, so it can effectively prolong the penetration time of water molecules in the film. With the increase in the number of layers, the degree of MgO crystallization decreased. The Al_2_O_3_/MgO composite film with a 3:2 Al_2_O_3_:MgO layer alternation type and a thickness of 100 nm achieves the optimal water vapor shielding ability, and the WVTR is 3.26 × 10^−4^ g·m^−2^·day^−1^ at 85 °C and 85% R.H. Further increase in the number of layers will produce defects in the film, resulting in a decline in the film shielding ability. In terms of optical transmittance, the transmittance of the composite film is extremely low in the range of 200–300 nm, but in the range of visible light, the 3:2 Al_2_O_3_: MgO layer alternation type has good visible light transmittance. Our work shows that Al_2_O_3_/MgO composite films prepared by FCVA technology have great potential in flexible TFE.

## Data Availability

The data presented in this study are available on request from the corresponding author.
